# Role of ultrasonography in detection of the localization of the nasoenteric tube

**DOI:** 10.1186/cc14471

**Published:** 2015-03-16

**Authors:** R Dagli, H Bayir, Y Dadali, T Tokmak, Z Erbesler

**Affiliations:** 1 Ahi Evran University Education and Research Hospital, Kirsehir, Turkey; 2 Abant Izzet Baysal University, Medical School, Bolu, Turkey

## Introduction

In this study, we aimed to determine the success rate of nasoenteric tube (NET) insertion into the postpyloric area by ultrasonography (USG) and compare it with the commonly used method, direct abdominal radiography.

## Methods

Patients admitted to an adult ICU between April and July 2014 with an indication for NET insertion for enteral feeding were included in the study after informed consent was given from patients' relatives. Nasoenteric feeding tubes were placed using the blind bedside method by a single anesthesiologist. Any motility stimulant agent was not used. The outside of the polyurethrane 8 F with unweighted NET (Bexen, Spain) and its guiding wire was lubricated with gel. The NET was inserted into the nostril after determination of the mouth- posterior ear-xiphoid distance and pushed on at least such a distance. Followed by auscultation of the gastric area and air infusion of 30 to 50 ml into the tube, the patient was positioned on their right side and the tube was advanced 20 to 30 cm more. Then the guiding wire inside the NET was removed. The patient was then brought to the supine position and NET was visualized by two radiologists simultaneously by M5 portable USG (Mindray, PRC), with a 3.5 MHz convex probe whether it passes through the postpyloric area or not. Localization of the tube was confirmed with abdominal radiography in all patients. During the first insertion of the NET, the ratios for inaccurate localization and correct placements through the postpyloric area were recorded and Results were compared with abdominal radiography.

## Results

In this study, the bedside blind method was used for NET insertion into 34 patients. Eleven of the tubes were detected passing through the postpyloric area by USG. In one case the NET could not be seen in the postpyloric area by USG, but it was detected in the postpyloric area by control abdominal radiography. In 22 patients, NETs were detected in the stomach with control abdominal radiography. Success for NET placement with the bedside blind method and USG imaging was 35% versus 91.6%, respectively.

## Conclusion

The success rate of the bedside blind method in the NET placement was low. It is clear that if any other placement techniques with high success rate will be applied, USG will be useful in a higher number of patients reducing the need for abdominal radiography.

